# Application of zero trust model in preventing medical errors

**DOI:** 10.3389/frhs.2024.1453804

**Published:** 2024-10-23

**Authors:** Nikhil Sood, Roop Parlapalli, Pranav Sharma, Rahul Kashyap

**Affiliations:** ^1^ Department of Medicine, Banner Health, Gilbert, AZ, United States; ^2^ Department of Medicine, Geisinger Community Medical Center, Scranton, PA, United States; ^3^ Department of Research, Global Remote Research Scholars Program, Princeton Junction, NJ, United States; ^4^ Department of Research, Well Span Health, York, PA, United States

**Keywords:** zero trust model, medical errors, patient safety, outcome, zero trust in healthcare

## Abstract

Medical errors can occur in many areas of healthcare, including hospitals, clinics, and surgery centers. They can result in negative consequences for patients and their loved ones. Over the years, different methods have been used to reduce medical errors. Zero Trust is an information security model that denies access to applications and data by default. Other industries have successfully used Zero Trust Model (ZTM), and it has been shown to improve outcomes. This editorial analyzes how the ZTM can be introduced to prevent medical errors in healthcare settings. ZTM application in healthcare could potentially revolutionize patient safety by tightly controlling and monitoring access to sensitive patient data and critical systems. By enhancing security measures, the ZTM could address the paramount concerns of patient data privacy and safety in healthcare. The zero-trust approach offers a potential solution by identifying consistent causes of errors and providing viable solutions to prevent their recurrence. In the era of worsening ransomware attacks on healthcare systems, the ZTM could also have enormous implications in other cybersecurity aspects. With this manuscript, the authors advocate for the broader application of ZTM across other facets of healthcare cybersecurity.

## Introduction

Medical errors have been identified as one of the leading causes of death in the United States. In 2019, unintentional injuries/accidents, which include medical errors, were the third leading cause of mortality after heart diseases and malignancies (1). Around one in every ten patients is harmed in health care, and more than 3 million deaths occur annually due to unsafe care. In low-to-middle-income countries, as many as 4 in 100 people die from hazardous care (2). Over 50% of harm is preventable, half of which is attributed to medications. Medical errors and adverse events among critically ill patients are associated with worse outcomes therefore interventions to minimize them are critical (3).

Zero Trust Model (ZTM) is an information security model whose default stance is that nobody is trusted. So, the model denies all access at the beginning. ZTM advocates these three core principles: all entities are untrusted by default, the least privileged access is enforced, and comprehensive security monitoring is implemented (4). In the past couple of years, the estimated cost of downtime in U.S. healthcare organizations caused by ransomware attacks was 14–16 billion U.S. dollars. The increased downtime and the non-availability of electronic health records have exponentially increased medical errors (5). In the background of increasing threats, including ransomware, implementing the ZTM is paramount, especially with user access to multiple devices and cloud-based utilization.

ZTM has been successfully implemented in various industries such as financial services, software companies, public sector companies, and healthcare systems, yet limited studies have been conducted in healthcare on utilizing the zero-trust model to prevent medical errors. This editorial highlights the principle of zero trust in healthcare settings to reduce medical errors, based on the framework provided by the US Cybersecurity and Infrastructure Security Agency. Implementing ZTM in healthcare holds immense potential to revolutionize patient safety. By tightly controlling and monitoring access to sensitive patient data and critical systems, the ZTM can significantly enhance security measures.

## Medical errors

A medical error is usually a preventable adverse effect of medical care. They create a serious public health problem that substantially threatens patient safety.

There are two major types of errors:

1. Errors of Omission occur as a result of actions not taken. Examples are not strapping a patient into a wheelchair or stabilizing a gurney before patient transfer.
2. Commission Errors occur due to the wrong action taken. Examples include administering a medication to which a patient has a known allergy or not labeling a laboratory specimen subsequently ascribed to the wrong patient (6).

There are four major types of medical errors: medication-related, surgical, healthcare-related infections, and falls. We discuss these and related issues.

## Medication-related errors

Medication error is widely accepted as the most common and preventable cause of patient injury. These are avoidable events that may cause or lead to inappropriate medication use or patient harm while the healthcare professional or patient controls the medication. Medication errors in hospitalized adults may cause damage, additional costs, and even death. Furthermore, there are an estimated 5.3 medication errors/100 prescriptions. These include missing doses (53%), dose errors (15%), frequency errors (8%), route errors (5%), and 1% were associated with adverse drug events (7). Adverse drug events have been shown to increase both the cost and the length of stay in community hospitals (8).

Some of the methods for preventing clinical errors are still in their infancy. The most promising technologies include electronic prescribing systems, diagnostic and clinical decision-making aids, and error-resistant systems. A study by Bates et al. found a significant reduction in medication errors with physician-computerized order entry (9). All these are still vulnerable during cyberattacks and ransomware attacks, further complicating the issue.

A systematic review of 65 studies involving 3,755 patients concluded that errors in prescription medication histories occurred in up to 67% of cases. These included commission and omission errors, incorrect dose, and incorrect frequency (10). Accurate and complete medication lists benefit patient care but are often unavailable. A recent Cochrane meta-analysis concluded that compared to usual care, medication reconciliation, computerized physician order entry (CPOE)/clinical decision support systems (CDSS), barcoding, feedback, and dispensing systems in surgical wards may reduce adverse drug events (ADEs), medication errors, or both (11).

## Surgical errors

Over 200 million surgical procedures are performed each year globally, and despite awareness of adverse effects, surgical errors continue to occur at a high rate. Surgical errors account for many adverse events, including near misses and adverse events leading to patient harm (12).

Implementing ZTM specifically in the contexts of medication dosing, operating room (OR) processes, and emergency room (ER) processes can significantly reduce medical errors:

A. **Multi-Factor Authentication (MFA):** Implementing MFA for accessing medication dosing systems, OR schedules, and patient records in the ER to ensure that only authorized personnel can input or modify data, reducing the risk of incorrect dosages or procedural errors (13).
B. **Role-Based Access Control (RBAC):** Implementing RBAC to ensure that staff can only access information and perform actions relevant to their role (14). For example, nurses may access patient charts for dosing, but only pharmacists can verify medication orders, and surgeons can update OR schedules.
C. **Real-Time Monitoring and Alerts:** Using real-time monitoring to track medication dispensing, OR activities, and ER procedures. Immediate alerts for deviations from standard protocols can help catch potential errors before they affect patient care (15).

## Healthcare-related infections

Healthcare-related infections add close to 35–43 billion dollars to the annual cost of healthcare in the United States (16). As many as 1 in 10 hospitalized patients may acquire a healthcare-related infection, increasing the hospital stay's complications, length, and cost (16, 17). These preventable harms can be minimized by educating personnel and staff involved in procedures ranging from simple surgical procedures such as urinary catheters to central line placements and protocols for ordering C diff testing and culture data.

## Falls

Falls are a common problem. Each year, more than one in four people over the age of 65 suffer a fall, and one-third of these falls cause injuries (18). In a healthcare setting, several factors may further increase the risk of falls, including using diuretics, blood loss, cluttered rooms, inadequate lighting, and altered mental status. Several interventions to reduce risk and increase patient safety are done in the hospital, including bed alarms and gait belts for elderly patients and placing call lights and personal things within patients’ close reach. Using the zero-trust model, it can be ensured that these best practices are followed and that all involved healthcare personnel follow established protocols properly.

Now, we discuss ZTM’s role in preventing medical errors in five major domains.

## Using zero trust model principles for prevention of medical errors

The US Cybersecurity and Infrastructure Security Agency recommended the ZTM framework for simplifying the complex healthcare delivery process into five domains—identity, devices, networks, applications, and networks and data (19). Using the principles of ZTM, as outlined in Table 1 below, shows its applicability in reducing medical errors and improving patient safety.

**Table 1:** Domains of zero trust model and medical error prevention.

Domains	Zero trust model (ZTM)	Medical error prevention and patient safety	Examples
Identity	Data access, specification of roles	• Important for designations, duties, roles, and responsibilities • Encouraging teamwork—Promoting psychological safety	• Timeouts before any procedures in the OR • Using Team- STEPPS framework—for unit-based teamwork on medical and surgical floors
Devices	Multi-Factor authentication Phishing resistance	• Modules and EHR training • Interface remodeling • BYOD-Bring your device policies and secure VPNs • Continuous validation and approval processes • Error prevention in entry and scanning—human design and systems thinking and remodeling	• Badges with Unique IDs • Second-factor authentications • Barcode scanning at the bedside • Updating hardware/devices • Ergonomic approach
Networks	Network segmentation, Traffic encryption, and management	• Interdisciplinary access and contributions from multi-disciplinary teams.	• Interfaces for different specialties and departments
Applications and workloads	Application threat protections, Secure application development and deployment workflow, application security testing	• Third-party access utilizations, validation, and approval process with constant updates • Failure Mode and Effect Analysis	• Quality Improvement projects and system improvement workflows • Scheduled IT and software updates with notifications and memos • Hardware maintenance updates with backup plans
Data	Data availability, access, encryption, categorization	• Dashboards and accessibility of near misses and adverse events • Morbidity, Mortality, and Improvement updates • Quality Improvement Projects with status updates	• Department-specific roles and designations for entry and safeguards • Patient safety dashboards • Health Informatics oversight

### Identities

The first domain to focus on is specifying the identity of the personnel involved in healthcare who have access to electronic health record systems and how they access secure networks and deliver healthcare. This means the roles and responsibilities of the individuals performing the clinical and nonclinical tasks must be specific. In complex healthcare systems, multiple personnel are involved, both directly at the bedside and remotely, including coding and billing staff, who are virtually connected, helping provide the best quality care.

ZTM helps by being intentional and focused regarding specifying the role and amount of access needed to perform those tasks. An AHRQ-published Team STEPPS tool could be helpful regarding time outs, huddles, briefs, and debriefs after elective procedures or hospital events (20). STEPPS is a teamwork system designed for healthcare professionals. It is a powerful solution to improving patient safety within an organization. It is an evidence-based teamwork system that enhances communication and teamwork skills among healthcare professionals. It aims to optimize performance among teams of healthcare professionals—enabling them to respond quickly and effectively to whatever situations arise. It has been shown to be beneficial in reducing falls in hospitalized patients (21).

### Devices

The second domain of the ZTM is the operability, security, and utility of the devices used in healthcare delivery. In addition to traditional devices used in healthcare, including computers, scanners, and radiological equipment, newer devices are integrated into individual patient care, such as wearable devices like fitness trackers. ZTM provides an approach to multifactor authentication and data dissection for specific users so that the system works seamlessly for all the other healthcare team members. This domain includes securing personal and office devices with antivirus software and upgrading the hardware to maintain strict confidentiality and security. The approach to preventing errors in health care has changed from individual to system level. Techniques to foster communication, reporting systems to identify latent errors, protocols to prevent medication errors, and practical training and simulation workshops are some of the strategies that can be adopted at the institutional level. Two-factor authentication (2FA) is a security measure that protects our digital lives. This significantly mitigates the risk of unauthorized account access and protects against phishing attacks (22).

### Networks

The third domain of focus for ZTM is securing networks to prevent a breach in one area from compromising the entire system, which could affect patient data, personnel information, or revenue. Healthcare systems are vulnerable to cybersecurity attacks that put patients at risk (23). The ZTM, with its workflow regulation, will help secure networks, minimizing damage and offering faster recovery (24). The software architecture of ZTM itself allows for the seamless operability of individual networks within complex healthcare systems so that it will not compromise services in other areas or departments.

### Applications and workloads

The fourth domain of ZTM is securing applications used in healthcare and regulating workloads. The complex healthcare system uses numerous applications across departments, and ZTM principles ensure that these applications are verified and validated at every step rather than at specific periods. The proactive approach of failure mode and effect analyses are performed, and regular assessments and simulations are to be better prepared for events with increased demand on technology and reliance on health information technology.

### Data

The fifth domain of ZTM is data. Every effort should be made to keep data as secure as possible and accessible to all involved members. With the emergence of technologies such as large language models (LLMs), generative AI, and artificial intelligence, protecting data is more critical than ever. Protected Health Information and data regulation without any data breaches are vital to the operation of any healthcare system. The healthcare information should be available for the right personnel with the right amount of information at the right time. Department-specific roles and designations should be assigned and followed. For example, physicians can view their billing patterns only with limited or no editing options; likewise, coding and billing staff can modify the billing data but not clinical information, which they can access but with no editing function. Many health systems have health informatics divisions that provide data to clinicians, researchers, administrative staff, and leadership for efficient patient care and health care delivery (25).

## Zero trust maturity models (ZTMM)

**Figure 1:**
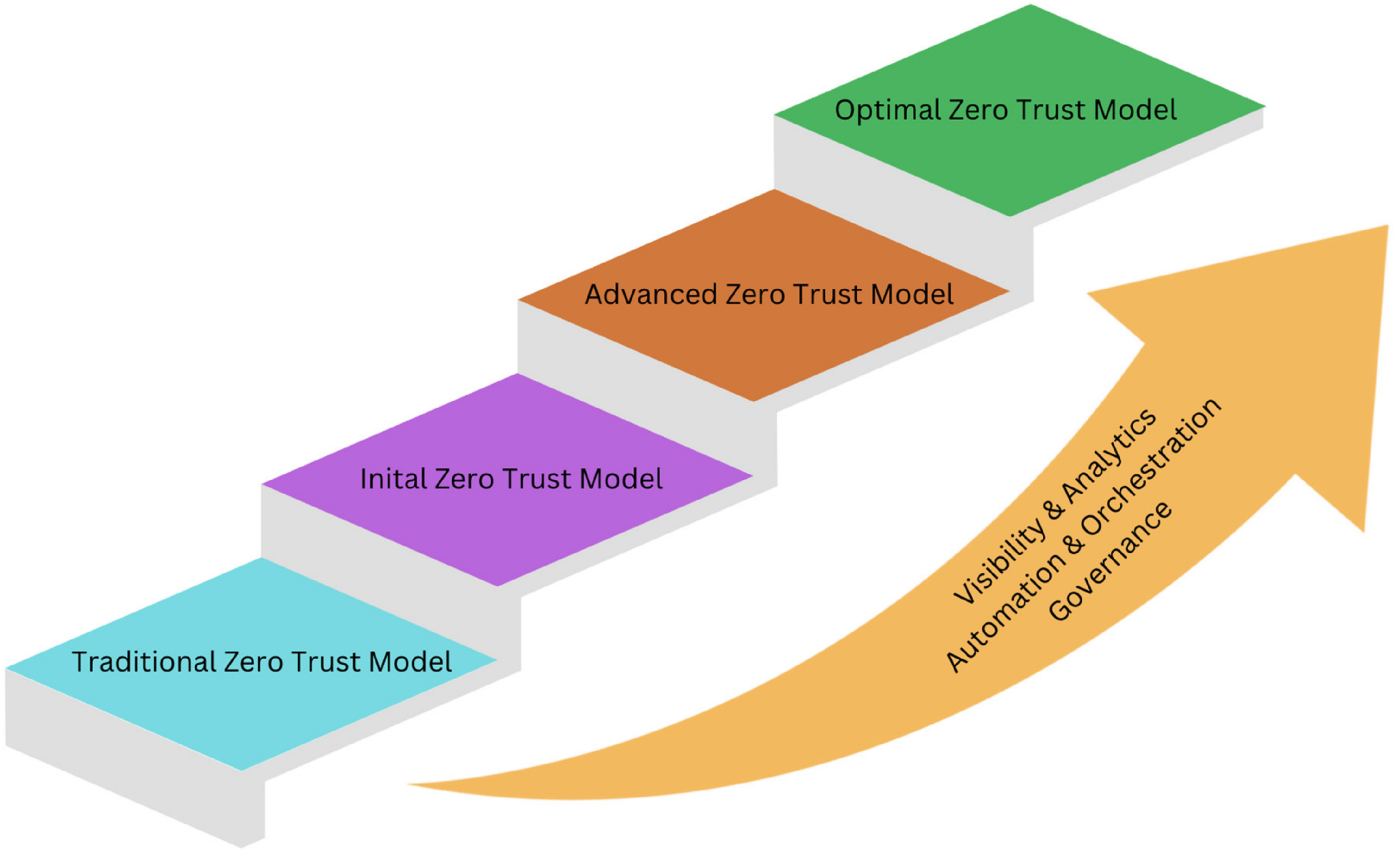
How to integrate zero trust model into healthcare system.

The ZTM is not a fixed model but a constant pursuit of improvement (26). Any healthcare system can utilize the principles of ZTM with its infrastructure and mature into advanced/optimal models (Figure 1). It depends on the extent of data visibility and analytics, orchestration and automation of different systems, and the governing policies guiding the organization. The Zero Trust Maturity Model (ZTMM) provides the framework for any healthcare system, from its existing health system to the optimal ZTM (27). The Optimal Zero Trust Maturity Model (Optimal ZTMM) embodies the epitome of this structure, known for its levels of transparency, analysis, automation, coordination, and governance across all zero trust realms. Traditionally, healthcare systems have depended on procedures, which makes them susceptible to inefficiencies and mistakes. Shifting towards a ZTMM bolsters security and effectiveness, offering a defense against emerging threats.

Guidance from the United States Cybersecurity and Infrastructure Security Agency (CISA) is available, and it provides a roadmap to achieving increased security and functionality of Zero-Trust Models (19, 24). The principles of ZTMM provide instructions for organizations to reach the optimal state where, despite evolving threats and technology, the number of errors is reduced. As the adoption of remote working and telehealth services has increased, the need for secure remote access and phishing-resistant multi-factor authentication has also increased.

In the Optimal maturity model, every domain of the ZTM operates at the highest standards. This includes the identities of personal devices used by healthcare professionals, operational networks, security protocols, applications, and healthcare data management. Institutional mission, values and priorities, organizational demands, and community needs drive policies and protocols for implementing ZTM into the Optimal ZTMM. This alignment ensures the delivery of safe, timely, effective, equitable, and patient-centered care, as the National Academy of Medicine advocates in its report “Crossing the Quality Chasm” (28).

## Addressing implementation challenges

1. **Cultural Shift:** Integrate zero-trust strategies with broader healthcare objectives to mitigate clinical and administrative staff pushback. Effective communication and collaboration across departments are essential. Executive support and leadership engagement are crucial for success, ensuring zero-trust initiatives reduce the risk of breaches that could impact patient care. Continuous education and training will foster a security-aware culture where every healthcare worker understands and upholds zero-trust principles.
2. **Continuous Monitoring:** Deploying advanced monitoring tools to provide real-time insights into network activity is essential for maintaining enhanced security and risk mitigation. These tools enable healthcare organizations to implement strict access controls and multi-factor authentication, significantly reducing the risk of unauthorized access and data breaches. By continuously monitoring network activity, healthcare systems can quickly identify and respond to potential threats, minimizing downtime and preventing medical errors. This proactive incident response ensures critical systems remain operational and available, contributing to uninterrupted and efficient patient care (29).
3. **Stakeholder Engagement:** Engaging all stakeholders, including clinicians, nursing staff, ancillary health care team members, IT staff, administrators, and patients, is crucial for implementing ZTM. Their feedback can help tailor security measures to meet the unique needs of the healthcare environment.
4. **Scalable Solutions:** Implementing scalable security solutions that can adapt to healthcare organizations’ growing needs is vital. Regular updates and reviews of security policies are necessary to keep pace with technological advancements and emerging threats.

The journey toward the Optimal maturity model of Zero Trust is complex, requiring a multifaceted approach encompassing technology, culture, and continuous improvement. However, the potential benefits—enhanced security, improved patient outcomes, and reduced operational risks—make it a compelling endeavor. As the healthcare industry evolves, embracing zero-trust principles will be essential in building resilient and secure healthcare systems.

The concept of zero-trust principles in healthcare settings has the potential to reduce medical errors significantly. By adopting a zero-trust approach, healthcare organizations can ensure that every access request is rigorously vetted, every transaction is meticulously monitored, and every anomaly is promptly investigated. This rigorous scrutiny can significantly reduce the risk of medical errors resulting from unauthorized access, data breaches, and system downtimes.

## Conclusion

Healthcare organizations can reduce the likelihood of errors caused by unauthorized personnel accessing or modifying sensitive information using the principle of least privilege access and limiting access to critical systems and patient data. Educating healthcare providers and maintaining a secure environment is essential to preventing medical errors and avoiding adverse health outcomes. Regular training sessions and awareness campaigns help build a culture of security consciousness within the organization. Multi-factor authentication, phishing resistance, network segmentation, traffic encryption, management application threat protections, and secure application development and deployment are some ways ZTM can be incorporated into healthcare systems. This comprehensive security framework prevents medical errors, promotes patient safety, and may help improve healthcare outcomes. Implementing a ZTM approach is an ongoing process that requires continuous monitoring, evaluation, and improvement to achieve an optimal state.

## Data Availability

The original contributions presented in the study are included in the article/Supplementary Material, further inquiries can be directed to the corresponding author.
